# Identification of missed viruses by metagenomic sequencing of clinical respiratory samples from Kenya

**DOI:** 10.1038/s41598-021-03987-1

**Published:** 2022-01-07

**Authors:** My V. T. Phan, Charles N. Agoti, Patrick K. Munywoki, Grieven P. Otieno, Mwanajuma Ngama, Paul Kellam, Matthew Cotten, D. James Nokes

**Affiliations:** 1 Virus Genomics, Wellcome Trust Sanger Institute, Hinxton, UK; 2 MRC/UVRI & LSHTM Uganda Research Unit, Entebbe, Uganda; 3 Epidemiology and Demography Department, KEMRI –Wellcome Trust Research Programme, Kilifi, Kenya; 4 School of Health and Human Sciences, Pwani University, Kilifi, Kenya; 5 Division of Infectious Diseases, Department of Medicine, Imperial College London, London, UK; 6 MRC-University of Glasgow Centre for Virus Research, Glasgow, UK; 7 School of Life Sciences and Zeeman Institute for Systems Biology and Infectious Disease Epidemiology Research (SBIDER), University of Warwick, Coventry, UK

**Keywords:** Infectious-disease diagnostics, Pathogens, Adenovirus, Influenza virus, Measles virus, Metagenomics

## Abstract

Pneumonia remains a major cause of mortality and morbidity. Most molecular diagnoses of viruses rely on polymerase chain reaction (PCR) assays that however can fail due to primer mismatch. We investigated the performance of routine virus diagnostics in Kilifi, Kenya, using random-primed viral next generation sequencing (viral NGS) on respiratory samples which tested negative for the common viral respiratory pathogens by a local standard diagnostic panel. Among 95 hospitalised pneumonia patients and 95 household-cohort individuals, analysis of viral NGS identified at least one respiratory-associated virus in 35 (37%) and 23 (24%) samples, respectively. The majority (66%; 42/64) belonged to the *Picornaviridae* family. The NGS data analysis identified a number of viruses that were missed by the diagnostic panel (rhinovirus, human metapneumovirus, respiratory syncytial virus and parainfluenza virus), and these failures could be attributed to PCR primer/probe binding site mismatches. Unexpected viruses identified included parvovirus B19, enterovirus D68, coxsackievirus A16 and A24 and rubella virus. The regular application of such viral NGS could help evaluate assay performance, identify molecular causes of missed diagnoses and reveal gaps in the respiratory virus set used for local screening assays. The results can provide actionable information to improve the local pneumonia diagnostics and reveal locally important viral pathogens.

## Introduction

Pneumonia is a leading cause of illness globally^1^ and determining the aetiologies of the disease would help case management, especially with regard to bacterial versus viral infections and decisions to use antibiotics as first-line therapeutic treatment. Polymerase chain reaction (PCR) methods are frequently used for viral diagnosis but costs and logistics limit the number of pathogens included in diagnostic panels. Additionally, mismatches in primers can cause missed diagnoses with evolving RNA pathogens^2,3^. Other viruses which are not included in the diagnostic panels due to costs may also contribute to missed diagnoses. Studies in Kilifi (coastal Kenya) to investigate viral aetiologies of pneumonia in paediatric patients and in the community identified respiratory viruses with these methods^4–9^; however, a proportion of samples collected from symptomatic children were negative for any respiratory viruses in the diagnostic screening panel.

We evaluated the ability of next-generation sequencing (NGS) to identify viruses missed by the diagnostic panel by evaluating samples from patients with respiratory disease which tested negative for common respiratory viral pathogens. A random priming (agnostic) NGS method was employed directly on clinical samples to detect viruses present in the samples by viral metagenomics^10,11^ without prior knowledge or specific PCR primers. The data generated from agnostic viral metagenomics of clinical samples were used to control the quality of the diagnostics (e.g. determine the presence of unanticipated viruses and variants of unexpected viruses on diagnostic panel), in order to help define the sensitivity of the diagnostic panels, reveal additional common respiratory pathogens, and improve future diagnostic assays for the region. These data will also help infectious disease clinicians to suspect potential causes that are not routinely included in the diagnosis if the clinical presentations warrant such a diagnosis.

## Results

### The detection of viruses

As shown in Table 1, viral NGS identified at least one syndrome-associated mammalian virus in 35 of 95 Kilifi County Hospital (KCH) inpatient samples (36.8%) and 23 of 95 household cohort (study investigating Who-Acquires-Infection-From-Whom, WAIFW) samples (24.2%), leading to an overall 30.5% “missed virus detection rate”. Among 58 samples that yielded a virus were six samples with mixed virus infections (5 in KCH patients and 1 in the household cohort). In the five KCH mixed infections, four were human rhinovirus A (HRV-A)/respiratory syncytial virus B (RSVB), HRV-A/Enterovirus D68 (EV-D68), Bocavirus/human rhinovirus B (HRV-B), or EV-D68/Coxsackievirus A16 (CV-A16) and one sample showed two distinct human rhinovirus C (HRV-C) strains. Overall, 64 viruses were identified; these were either missed viruses (viruses included in the standard diagnostic panel but the assay was negative; n = 38) or unexpected viruses (viruses not part of the standard diagnostic panel; n = 26).

**Table 1:** Detected respiratory viruses, sample source and our explanation for diagnostic failure.

Virus species	Virus family	KCH cohort	Household cohort	Grounds for diagnostic failure
HRV-A	Picornaviridae	4	4	Primer/probe mismatch
HRV-B	Picornaviridae	3	4	Primer/probe mismatch
HRV-C	Picornaviridae	11	2	Primer/probe mismatch
HMPV	Pneumoviridae	4	2	Primer/probe mismatch
RSVB	Pneumoviridae	3	0	Primer/probe mismatch
HPIV-1	Paramyxoviridae	0	1	Primer/probe mismatch
DENV-2	Flaviviridae	1	0	Not in panel
HHV-5	Herpesviridae	2	0	Not in panel
HSV-1	Herpesviridae	2	0	Not in panel
Bocavirus	Parvoviridae	1	3	Not in panel
Parvovirus B19	Parvoviridae	1	0	Not in panel
Echovirus E1	Picornaviridae	0	1	Not in panel
EV-D68	Picornaviridae	4	0	Not in panel
CV-A16	Picornaviridae	1	0	Not in panel
CV-A24	Picornaviridae	0	6	Not in panel
Parechovirus	Picornaviridae	0	1	Not in panel
Poliovirus	Picornaviridae	1	0	Not in panel
Rubella	Matonaviridae	2	0	Not in panel
Total virus identified		40	24	

### Missed viruses

The number of respiratory viruses (with contigs ≥ 1000 nt) that had been missed by the standard diagnostic panel included 28 human rhinovirus (HRV) in 27 samples (14.2%; 27/190), one human parainfluenza virus 1 (HPIV-1) (0.5%; 1/190), three RSVB (1.6%; 3/190), and six human metapneumovirus (HMPV) (3.2%; 6/190) (Table 1). For these missed viruses, nucleotide mismatch between the diagnostic primers and probes and the viral target sequence were identified, potentially accounting for detection failures (see below).

### Analysis of primer mismatches

**Figure 1:**
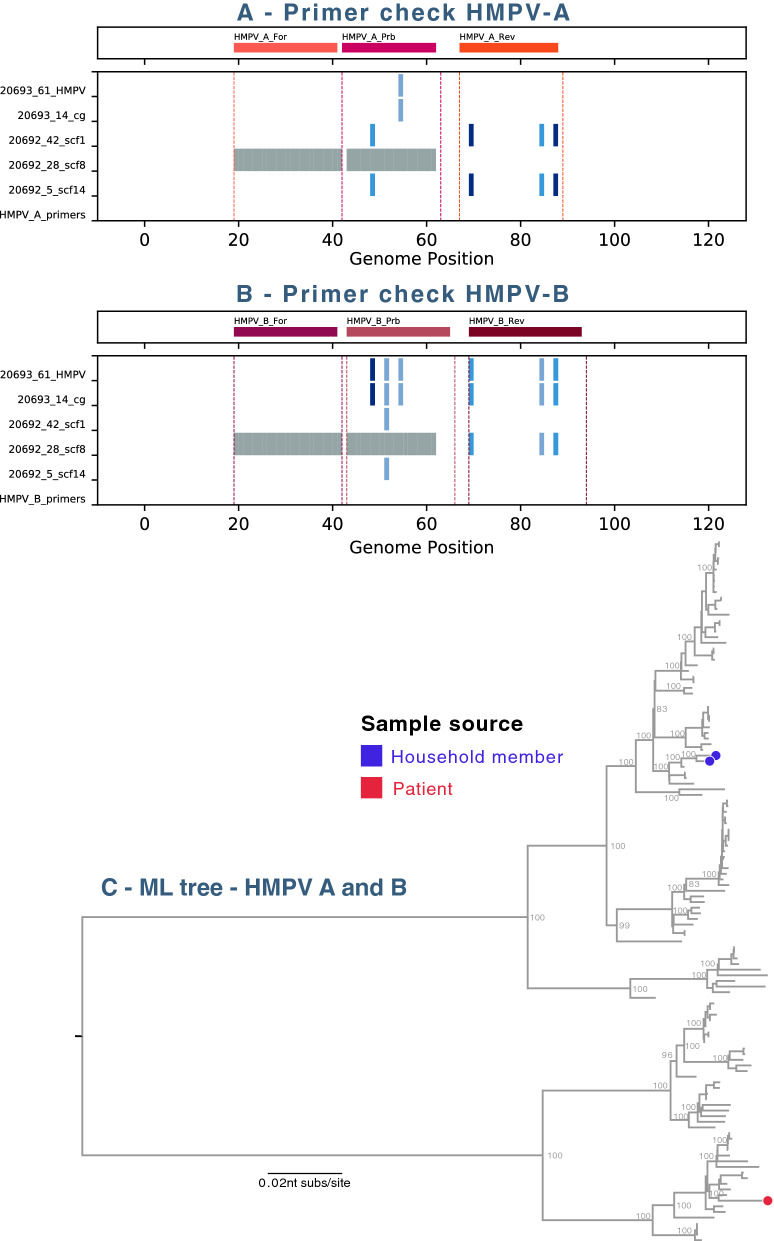
Human metapneumovirus (HMPV) identified in the study. (**A**) The diagnostic primers and probe target sites in the Kilifi HMPV genotype A genomes and contigs were examined. All viral contigs from each virus family or type were aligned using MAFFT^25^, and the alignment was trimmed to a 100–200 nt region surrounding the primer and probe target sites. Nucleotide differences between the expected primer and probe target sites and the actual contig sequences were identified and plotted in shades of blue and gaps in contig sequences were indicated in grey. (**B**) As in (**A**) but for HMPV genotype B. (**C**) Maximum-likelihood (ML) phylogenetic tree of HMPV genomes. Local strains on the phylogenetic tree were indicated by circles coloured in blue indicating household member and in red indicating KCH patients. The tree was mid-point rooted for clarity and horizontal branch lengths were drawn to the scale of nucleotide substitutions per site. The tree comparing local HMPV genomes to global genomes suggested that the local HMPV belonged to genotype A2 and B1.

**Figure 2:**
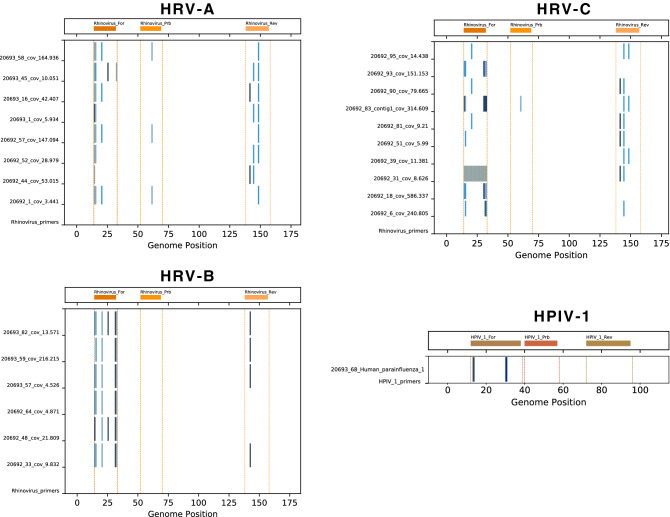
Diagnostic primers and probes check for human rhinoviruses (HRV) and human parainfluenza virus identified from the study. The diagnostic primers and probes target sites in the Kilifi HRV-A, HRV-B, HRV-C and HPIV-1 genomes were examined (see Fig. 1 legend for detailed methods). For each contig, nucleotide changes from the expected target sites were indicated by vertical blue lines, gaps in the sequence were indicated by grey bars.

Likely causes of missed diagnoses are mismatches between the primer/probes and viral target sites. For all viral contigs ≥ 1000 nt, if the virus family was part of the diagnostic panel, target sites were examined for differences from the primer/probe. For HRV-A, HRV-B, HRC-V, HMPV and HPIV-1, a number of nucleotide changes were observed in target sites and most were consistent with failed or suboptimal diagnostic tests (Figs. 1, 2). For the RSVB genomes detected, there were nucleotide changes in the probe targets; an updated panel of primers/probe was recently developed and used successfully^1^.

### Unexpected viruses

An advantage of the agnostic viral NGS is the ability to detect viruses present in a specimen without a prior knowledge of virus genome sequence for primer design. In the 190 samples, 26 unexpected viruses from five families of viruses were identified, none were included in the standard diagnostic panel (Table 1). Most unexpected viruses (50%; 13/26) were *Picornaviridae*, genus *Enterovirus*, species A (CV-A16, n = 1), species B (Echovirus E1, n = 1), species C (CV-A24, n = 6 and human poliovirus 2 strain Sabin, n = 1), and species D (EV-D68, n = 4). The *Parvoviridae* Human bocavirus (HBoV) and parvovirus B19 (B19) were identified in four and one sample (Table 1). Rubella virus (RVi) was detected in two KCH paediatric patients with very different clinical presentations (further details below).

### Human metapneumovirus

Human metapneumovirus (HMPV) infection is frequent in young children^12^ and was identified in six samples (Table 1). The diagnostic primer targets in the genomes showed mismatches (Fig. 1, panels A, B) that could explain the missed HMPV diagnostics. A maximum-likelihood phylogenetic tree comparing the HMPV complete genomes from the local strains to global circulating strains showed the two HMPV from the household cohort were genotype A2; the HMPV from the KCH patient was genotype B1, closest to strains KC562240 (A2) and KF530179 (B1) from Australia in 2003 (Fig. 1, panel C). The reported HMPV sequences were also compared with local Kenya HMPV short sequences available from GenBank, all fell into similar lineages (Supplementary Fig. S1, panel B) and were likely missed because of primer mismatch rather than because they were a new lineage.

### *Enterovirus* genus

Among 64 viruses detected, the majority were from the *Enterovirus* genus, *Picornaviridae* family (N = 42; 66%). Apart from HRV (N = 28), viruses from the *Enterovirus* species were not included in the routine screening.

### *Rhinovirus* species, *Enterovirus* genus

The most abundant *Enterovirus*es identified were *Rhinovirus* species A, B and C, with 28 complete or partial genomes identified. All three sets of diagnostic primers used at the time showed multiple mismatches with the genome target sites that could account for the 28 missed HRV cases (Fig. 2). A high diversity of circulating HRV has been noted in this region^6,13^ as shown in phylogenetic trees comparing local HRV identified from this study with global HRV genomes (Supplementary Fig. S2).

### *Enterovirus* A species (CV-A16), *Enterovirus* genus

Coxsackievirus A16 (CV-A16), enterovirus 71 (EV-71) and several additional *Enterovirus* species are associated with hand, foot and mouth disease (HFMD)^14^. The 22-month old patient infected with CV-A16 was hospitalized at KCH with pneumonia, but presented no clinical HFMD symptoms, and was discharged home after 3 days. Phylogenetically, the patient’s CV-A16 virus genome was closely related to a CV-A16 strain identified from an Ethiopian child in April 2016 (Supplementary Fig. S1, panel A)^15^.

### *Enterovirus* C species (CV-A24), *Enterovirus* genus

**Figure 3:**
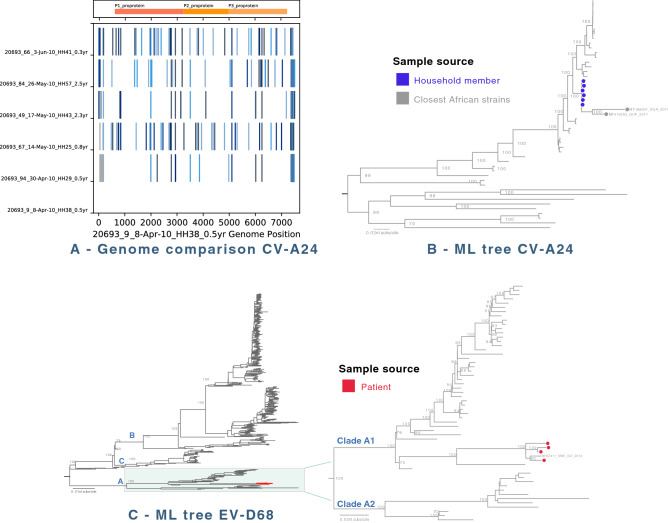
(**A**) Genome comparison of identified human coxsackievirus A24 (CV-A24) from the study. Kilifi CV-A24 genomes were examined and compared against the earliest Kilifi CV-A24 genome identified (20693_9, sample collected on 8 April 2010). For each genome, nucleotide changes from the first CV-A24 genome (20693_3) were indicated by vertical blue lines, gaps in the sequence were indicated by grey bars. (**B**) Maximum-likelihood phylogenetic tree of CV-A24 genomes. The ML tree compared 6 Kilifi CV-A24 genomes (all from household cohort, indicated as blue circles) to global genomes. The tree was mid-point rooted for clarity and horizontal branch lengths were drawn to the scale of nucleotide substitutions per site, and significant bootstrap values were shown for major nodes. (**C**) Human enterovirus D68 (EV-D68) maximum-likelihood phylogenetic tree. Maximum-likelihood phylogenetic tree were inferred comparing 4 Kilifi EV-D68 genomes (all from KCH pneumonia patients cohort, highlighted in red) to global genomes. The tree was mid-point rooted for clarity and horizontal branch lengths were drawn to the scale of nucleotide substitutions per site, and significant bootstrap values were shown for major nodes. These four local EV-D68 viruses belonged to clade A1, as shown in the zoomed out tree.

Coxsackievirus A24 (CV-A24) was identified in six samples, all from a 2-month period (8 April thorugh 3 June 2010) in the household study (Fig. 5). The six infected individuals were aged 8.5 to 33 months, and came from different households. None of these children presented with conjunctivitis, one had diarrhea and all had rhinorrhea. The identified CV-A24 genomes showed 12 to 146 nt differences and very few shared SNPs (Fig. 3, panel A), suggesting that the viruses were not directly transmitted between the 6 individuals. The samples were selected to cover as many households as possible over the entire cohort time period, thus the observed diversity may reflect a much larger outbreak that would account for the number of nucleotide changes. This is also consistent with the monophyletic phylogeny for the six genomes (Fig. 3, panel B). When analyzed with all available CV-A24 genomes from GenBank, the local CV-A24 sequences formed a monophyletic group closest to sequences from Uganda (GenBank MF189567) and French Guiana (GenBank MF419263) which were associated with ocular inflammation or acute haemorrhagic conjunctivitis (AHC) in 2017^16^ (Fig. 3, panel B).

### *Enterovirus* C species (human poliovirus), *Enterovirus* genus

The detection of human poliovirus type 2 was likely due to viral shedding after oral poliovirus vaccination. The child in whom this isolate was detected was 6 weeks old at the time of sampling, and had received a dose of oral polio vaccine (OPV) 4 days prior. The child was hospitalised with cough and difficulty breathing, and was discharged home after 3 days. The human poliovirus 2 genome obtained was identical to the vaccine strain Sabin 2 (GenBank AY184220). Health authorities were informed about this finding.

### *Enterovirus* D species (EV-D68), *Enterovirus* genus

Four EV-D68 were identified in the hospitalised cohort over a 5-month period (8 April through 318 July 2010). Two of these patients were co-infected with additional Enterovirus strains, CV-A16 or HRV-A. The four children with EV-D68 were hospitalised with severe pneumonia for 3–6 days, and were discharged with no report of neurological symptom.

Phylogenetic analysis (Fig. 3, panel C) suggested that the four Kilifi EV-D68 viruses were clade A1 and closely related to a strain identified from a respiratory patient in 2014 in Sweden (GenBank MH674114) and to a Canadian EV-D68 strain in 2014 (GenBank KP455258).

### *Matonaviridae* family, *Rubivirus* genus (Rubella virus)

Rubella is a contagious typically mild disease caused by rubella virus (RVi), a single-stranded RNA virus in the *Matonaviridae* family, *Rubivirus* genus, infecting people of any age. However, primary RVi infection during the first trimester of pregnancy may result in congenital rubella syndrome (CRS) or miscarriage. Common sequelae of CRS include deafness, glaucoma and retinopathy and heart defects. Rubella infections can be prevented by highly effective rubella vaccine. In Kenya, national rubella vaccination was not implemented until October 2016 and there was no surveillance of RVi prevalence or CRS incidence^17^.

**Figure 4:**
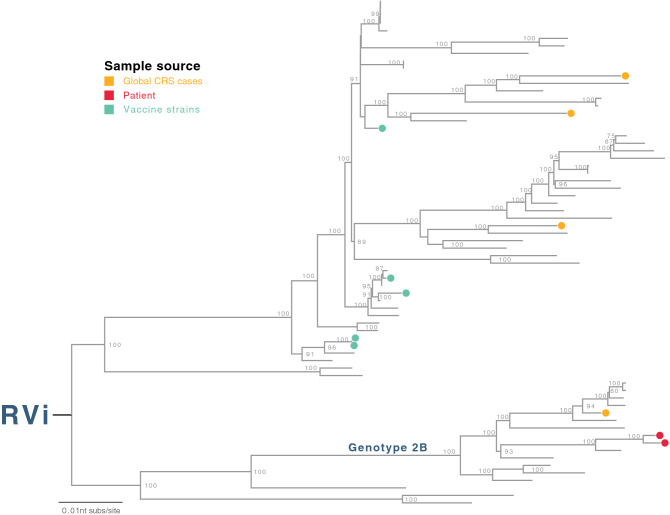
Human rubella virus (RVi). Maximum-likelihood phylogenetic tree was inferred comparing two RVi genomes (all from KCH pneumonia patients cohort, indicated as red circles) to global genomes. The global RVi strains identified in confirmed CRS cases were indicated as orange circles in the tree, and RVi vaccine strains were indicated as green circles. The tree was mid-point rooted for clarity and horizontal branch lengths were drawn to the scale of nucleotide substitutions per site, and significant bootstrap values were shown for major nodes.

RVi was detected in two KCH patients through this NGS study (Table 2). They were 10 days and 27 days old at the time of hospitalisation, and had admission diagnoses of neonatal sepsis. Their hospital admissions occurred 5 weeks apart. Phylogenetic analysis on the complete RVi genomes compared with all available RVi genomes available from Genbank indicated that the two Kilifi RVi genomes were similar (60nt differences, 99.4% identity) and belonged to the same genotype (genotype 2B, Fig. 4). Rubella is not routinely screened for or suspected in respiratory infections or neonatal sepsis. Identification of RVi in two neonatal patients in the context of absent or low vaccination coverage in LMIC settings, would alert clinicians to consider this virus in their diagnoses.

**Table 2:** Infection features of two rubella cases.

Features	Case A	Case B
Gender	Female	Female
Place of birth	Home	Home
Age at admission	10 days	27 days
Discharge	2 days after admission	4 days after admission
Discharge type	Death	Alive
Discharge diagnosis	Neonatal sepsis and haemorrhage	Neonatal sepsis
Conscious level	Lethargic	Normal
Arrest type	Respiratory	
Arrest CPR	Bag and mask	
HDU admission	Yes	No
Additional comments	Meningitis with congenital cataracts	
MMR vaccination	No	No

*CPR* cardiopulmonary resuscitation, *HDU* high dependency unit.

### *Parvoviridae* family

We identified parvovirus B19 (B19) in a 5-month old hospitalised patient with very severe pneumonia and anaemia. Tests for malaria were negative, and the patient was discharged after 3 days. The identified B19 virus genome belonged to genotype 1A, similar to other global B19 sequences as shown in the phylogenetic tree comparing the local B19 genome to global sequences (genotype 1A, Supplementary Fig. S1, panel C).

Human bocavirus (HBoV) type 1 was identified in a hospitalised child with malnutrition, severe pneumonia and diarrhoea, and in three children with upper respiratory infections from different households. These 4 HBoV1 genomes clustered in 2 sub-lineages within genotype 1 when compared with all global sequences as shown in Supplementary Fig. S1, panel D.

### Other viruses

Viruses detected at low frequency included HPIV-1 (one case), human parechovirus (one case), human herpesvirus 5 (HHV-5; two cases), human herpes simplex virus (HSV-1; one case), Dengue Virus type 2 (DENV-2; one case) and echovirus E1 (one case).

### Detection timeline

**Figure 5:**
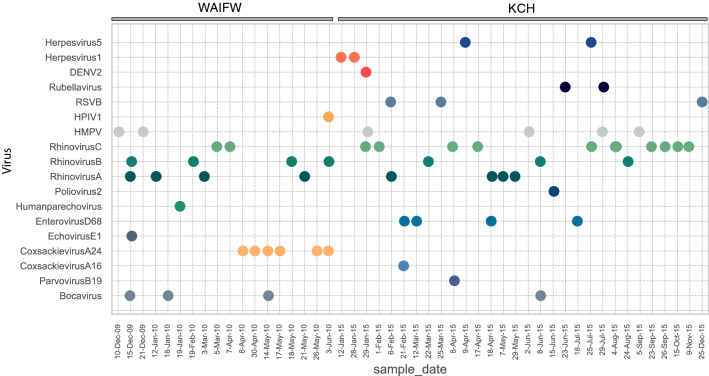
The timeline of viruses identified in the household cohort and KCH pneumonia patients. The viruses detected in the study (by row) were plotted against the date from which samples were collected (by column). Each virus was presented as a different colour. All the positive samples from household cohort were from 10 December 2009 to 3 June 2010, while positive samples from KCH patients were from 12 January 2015 to 25 December 2015.

The date of collection of specimens that were test negative using the routine viral panel assay, and their NGS viral detection results, are plotted by time (Fig. 5). The various HMPV, HRV-A, HRV-B and HRV-C positive samples are distributed throughout the observation period and occurred in both study groups (KCH and WIAFW). CV-A24 and EV-D68 positive samples were detected over discrete time periods (2 months in 2010 and 5 months in 2015, respectively) as mentioned above. The other observed viruses were too few to make strong conclusions about their temporal distribution.

## Discussion

Respiratory infections are a leading cause of morbidity and mortality worldwide, sensitive and accurate viral diagnostics are crucial for guiding clinical care. In this study, we investigated clinical respiratory samples from a single location in Kenya that had failed to return a diagnosis with the local PCR diagnostic panel. We used randomly primed (unbiased) metagenomic sequencing to increase the viral detection potential. The objectives of this study were twofold. Firstly, we were interested in the number of missed virus diagnoses in respiratory cases. Secondly, we sought respiratory infections in Kilifi whose agents were not included in the routine screening panel. Here we have demonstrated the utility of direct deep sequencing of clinical respiratory samples to identify virus genomes circulating in a resource-limited country. When applying this simple strategy of random-primed viral NGS in respiratory samples testing negative in local diagnostic panels, viral sequences were identified and the approach revealed several categories of missed diagnostics. *Category 1* The virus was in the diagnostic panel but locally circulating strains differed at diagnostic primer/probe sites. This is remedied by updating the diagnostic panel with locally appropriate primers, as being done recently^2^. *Category 2* The virus was detected but below the cut-off for a positive diagnosis. The current cut-off for positive diagnosis is PCR Ct < 35.0; yet the assays did not show Ct-values of negative diagnoses, hence limiting the interpretation of findings here. Further studies are needed to determine assay sensitivities to update positive/negative cutoffs. *Category 3* The virus was not in the diagnostic test panel. Due to practical constraints, it is not feasible to include all potential pathogens in a diagnostic panel, however findings from NGS analyses may support the decision to modify diagnostic panels accordingly. A limitation of this NGS approach is the threshold below which a virus does not yield identifiable sequences. Although 30.5% of the samples returned a viral diagnosis, 69.5% failed to yield a classifiable viral sequences. We expect that future improvements in NGS methods will increase the fraction of new diagnoses allowed by these methods.

The unexpected/not-tested viruses (i.e. rubella virus, poliovirus, EV-D68), providing additional possible causative agents explaining the symptoms and could help in the local guidelines and policy for disease management and practice. Common viruses were found that had been missed by diagnostics (e.g. HMPV, HRV and RSV). In many cases these detected virus sequences showed nucleotide changes in the primer and/or probe target sites that may account for the missed detection. Although rhinoviruses are the most common virus diagnosis in the Kilifi setting, they were frequently missed by the diagnostics^7,18^. Co-infections with multiple viruses were also detected which may also account for disease severity. Finally, the methods yielded complete or nearly complete genome sequences for respiratory viruses circulating in Kilifi providing a valuable sequence resource for improving local PCR diagnostic assays.

Although viral NGS would be expensive to apply for all diagnostics, the data from this study can inform an optimum pace of applying agnostic viral NGS to improve local diagnostics. The concept of the idea is illustrated in Supplementary Fig. S3. We expect declining diagnostic sensitivity over time (Supplementary Fig. S3, red dashed lines) due to virus evolution with altered primer target sequences, movement into the region of undetectable variants or viruses not on the diagnostic panel. Each round of NGS would result in a revised diagnostic panel, adjusted for local sequence variation and new viruses (“reset sensitivity”, Supplementary Fig. S3).

The data and analyses presented here provide a description of circulating respiratory viruses from two cohorts (severe pneumonia from hospital admissions and mild respiratory infections from a household cohort) from one region in coastal Kenya. Albeit small sample size and from a single location, the study included patients with a wide range of symptoms ranging from runny nose, sneezing to severe and very severe pneumonia, with all case types failing to yield a diagnosis for an aetiological pathogen. The study setting and methodologies, i.e. the combination of agnostic viral NGS on samples that had also been subjected to the local viral diagnostic panel, are the strength of the study. This combination allowed us to make important conclusions about the number and type of viruses missed by the local viral diagnostic panel and therefore provides useful information for improving local viral diagnostics.

## Materials and methods

### Study location and sample selection

Samples were randomly chosen from an acute respiratory disease surveillance at Kilifi County Hospital (KCH; a primary care and referral hospital)^5^ and a household cohort investigating Who-Acquires-Infection-From-Whom (WAIFW)^9^ from Kilifi, coastal Kenya. The hospital surveillance enrolled paediatric patients presenting with severe or very severe pneumonia based on clinical symptoms as previously defined^4,5^, and included children aged 1 day to 59 months and excluded babies with neonatal tetanus. For the KCH surveillance, naso- and oropharyngeal swab (NP/OP) samples were collected as soon as possible after hospital admission as previously described^19^. Ninety-five samples which had tested negative for the 15 respiratory viruses (see “Standard diagnostic panel” section) were randomly selected from January to December 2015 and represent the severe spectrum of respiratory infections in this study.

For the household cohort, members of households in a rural coastal in Kilifi were enrolled and an NP swab was collected from all members irrespective of respiratory symptoms at regular twice-weekly visits from December 2009 to June 2010^9^. Ninety-five samples from household members with symptoms of upper respiratory tract infection, which had tested negative for viral pathogens diagnosis, were chosen and represent the mild spectrum of respiratory infections in this study.

### Standard diagnostic panel

All samples were screened by multiplex real-time PCR for 15 respiratory virus targets^9,18,20^: respiratory syncytial virus (RSVA and B), influenza virus A, B, and C, human rhinovirus (HRV), human coronavirus (OC43, NL63 and 229E), adenovirus (AdV), human parainfluenza virus (HPIV1–4), and human metapneumovirus (HMPV). Primers, probes and target genes used for the PCR assays are summarised in Supplementary Table S1. Samples were considered positive when PCR cycle threshold (Ct) was < 35.0 for any of the 15 virus targets. For KCH samples, RSV antigen was determined using a direct ImmunoFluorescent Antibody Test (IFAT) (Light Diagnostic™ RSV DFA kit, Chemicon, Millipore Corporation, USA)^5^. The multiplex real-time PCR with or without the IFAT assay is referred to as “the standard diagnostic panel” throughout the manuscript.

### Sample preparation and agnostic deep sequencing

Total nucleic acid extraction and dsDNA conversion were performed as previously described^21^. Briefly, the method includes centrifugation and DNase treatment to remove free non-encapsidated DNA, reverse transcription with non-ribosomal random hexamers avoiding rRNA targets followed by sequencing on Illumina HiSeq 2500, generating 2–3 million 250 nt paired-end reads/sample.

### De novo assembly and identification of total viral genomes

Quality controlled reads (median Phred > 35, read length ≥ 175 nt, using QUASR^22^) were de novo assembled (SPAdes v.3.10^23^). Virus contigs were identified with UBLAST^24^ using virus family protein databases. Final quality control of genomes included checking open reading frames (ORFs), and comparison with reference sequences retrieved from GenBank. Stringent criteria for calling a sample positive for required greater than or equal to 1000 nt (or largest viral segment for segmented viruses).

### Phylogenetic construction

Global reference sequences were retrieved from GenBank, coding regions from reference and assembled genomes were extracted, and aligned using MAFFT^25^ and manually checked in AliView^26^. Maximum-likelihood phylogenetic trees were constructed in RAxML^27^ under the Generalised Time Reversible model (GTR) with 100 pseudoreplicates. Bootstrap values of ≥ 70% were considered statistically significant and were shown for major nodes. Tree was visualised in FigTree v.1.4.3 (http://tree.bio.ed.ac.uk/software/figtree). The tree was mid-point rooted for clarity and horizontal branch lengths were drawn to the scale of nucleotide substitutions per site.

### Ethical approval

The study was approved by the Kenyan Medical Research Institute Scientific and Ethics Review Unit (KEMRI-SERU) and the Coventry Research Ethics Committee (United Kingdom), and all methods were performed in accordance with the relevant guidelines and regulations by KEMRI-SERU and the Coventry Research Ethics Committee. Written informed consent was obtained for all eligible participants before sample collection. For children (< 18 years), informed consent was given by parents or guardians.

## Supplementary Information

Supplementary Legends.

Supplementary Information 1.

## Data Availability

Viral genome sequences from this study were deposited in GenBank (accession numbers MK989713–MK989765, Supplementary Table S2).
