# Supplementary material for: Identification of missed viruses by metagenomic sequencing of clinical respiratory samples from Kenya
Source: Sci Rep. 2022 Jan 7;12:202. doi: 10.1038/s41598-021-03987-1 (PMC8742071; doi:10.1038/s41598-021-03987-1)
Supplement: Supplementary file 1 — Supplementary Legends. [file 41598_2021_3987_MOESM1_ESM.docx]

**Supplementary Figure S1. Maximum-likelihood phylogenetic trees of CV-A16 (A), HMPV (B), B19 (C) and HBoV (D).**

**Panel A -** Maximum-likelihood phylogenetic tree was inferred comparing the Kilifi CV-A16 genome identified from a KCH patient (indicated as red circle) to global genomes. **Panel B -** Maximum-likelihood phylogenetic tree was constructed for HMPV partial sequences, comparing Kenyan HMPV sequences from this study (from KCH patients, indicated as red circle and from household members, indicated by blue circles) with other Kenyan HMPV sequences (indicated as orange circles) to global HMPV strains (blue circles). **Panel C -** Maximum-likelihood phylogenetic tree was constructed for B19 comparing the Kilifi viral genomes (identified from a KCH patient, indicated as red circle) to global reference viruses. **Panel D -** Maximum-likelihood phylogenetic tree was inferred for HBoV, comparing the Kilifi viral genomes (from KCH patients, indicated as red circle and from household members, indicated by blue circles) to global reference viruses. All trees were mid-point rooted for clarity, horizontal branch lengths were drawn to the scale of nucleotide substitutions per site, and significant bootstrap values were shown for major nodes.

**Supplementary Figure S2. Phylogenies of human rhinoviruses (HRV).** Maximum-likelihood phylogenetic trees were constructed comparing Kilifi HRV-A (Panel A), HRV-B (Panel B) and HRV-C (Panel C) genomes to global genomes. Local strains on the phylogenetic tree were indicated by circles coloured in blue indicating household cohort and in red indicating KCH patients. All trees were mid-point rooted for clarity, horizontal branch lengths were drawn to the scale of nucleotide substitutions per site, and significant bootstrap values were shown for major nodes.

**Supplementary Figure S3. Estimate of optimum viral NGS rate.** The estimated viral detection sensitivity ((detected virus/total virus)*100) over time was plotted (red dashed lines). A threshold of 75% sensitivity is indicated (black dotted line). Under these estimated conditions, if viral NGS occurs at week 90 and primer update is completed by week 120 the detection sensitivity > 75% could be maintained.
