# Supplementary material for: Identification of missed viruses by metagenomic sequencing of clinical respiratory samples from Kenya
Source: Sci Rep. 2022 Jan 7;12:202. doi: 10.1038/s41598-021-03987-1 (PMC8742071; doi:10.1038/s41598-021-03987-1)
Supplement: Supplementary file 2 — Supplementary Information 1. [file 41598_2021_3987_MOESM2_ESM.pdf]

Supplementary Figure S1

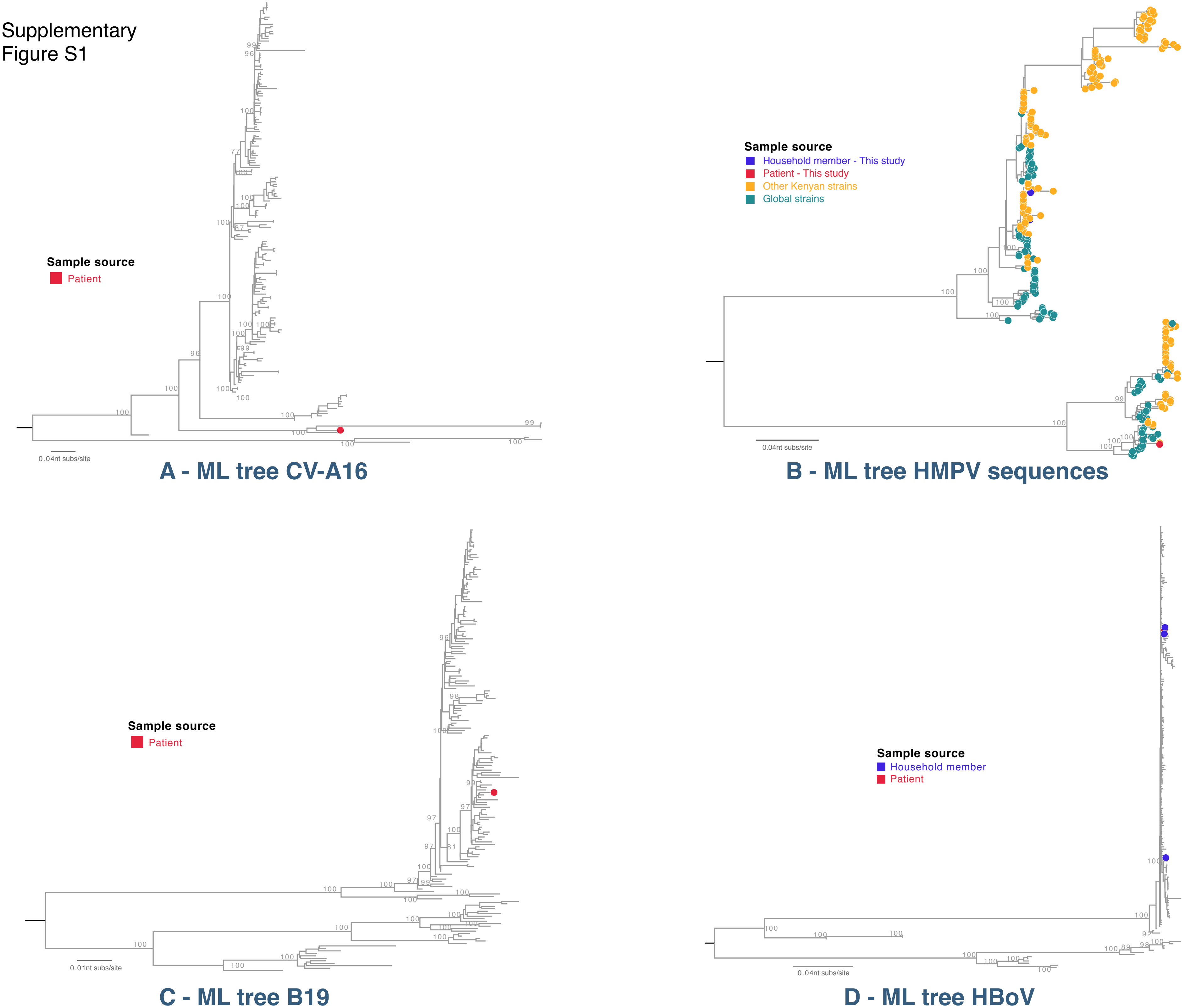

Supplementary Figure S2

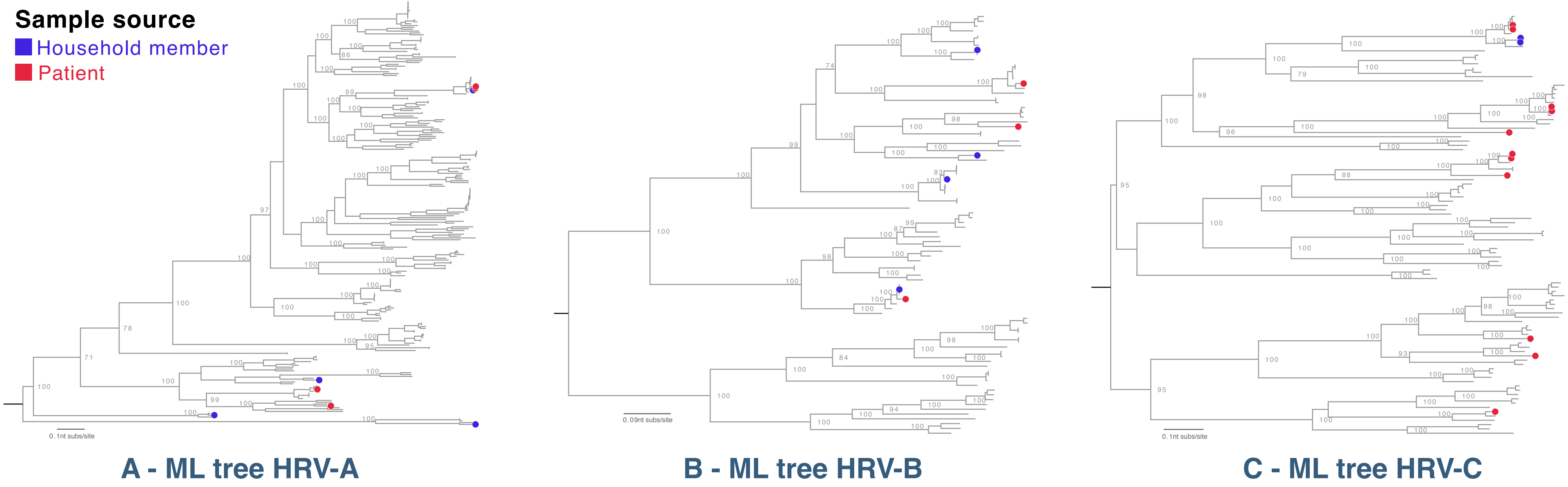

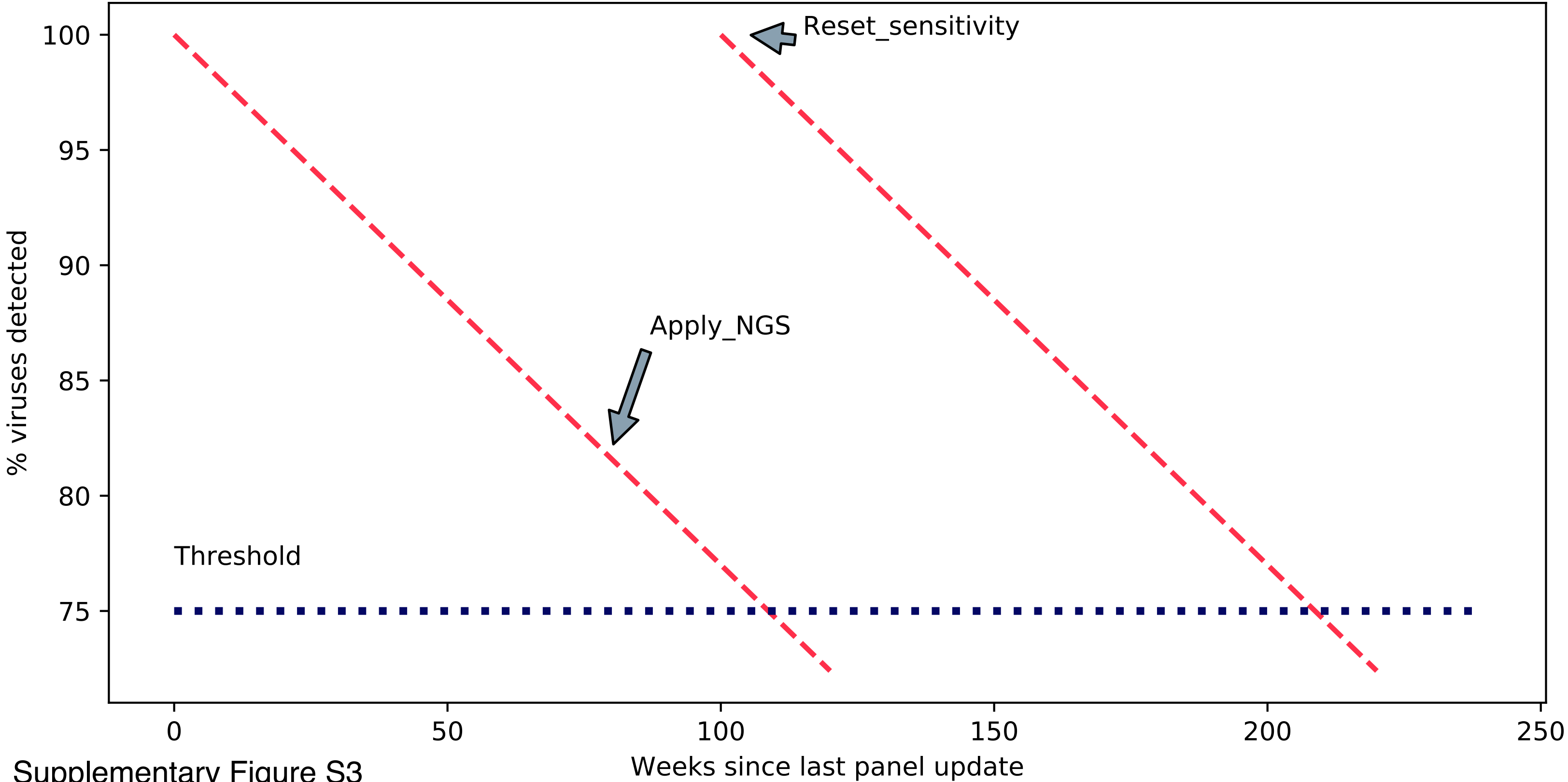

Supplementary Figure S3

Supplementary Table 1. List of diagnostic primers and probes used in the local respiratory viruses screening assays.

Target #	Organism	Target	Sequence (5' to 3')	5' Modification	3' Modification	Reference
1	Influenza A	Matrix	F, AAGACAAGACCAATYCTGTCACCTCT	-	-	(1) Gunson et al, 2010
			R, TCTACGYTGCAGTCCYCGCT	-	-	
			P, TYACGCTCACCGTGCCCAGTG	FAM	BHQ1	
2	Influenza B	NS	F, ATGATCTTACAGTGGAGGATGAAGAA	-	-	(2) Gunson et al, 2011
			R, CGAATTGGCTTTGRATGTCCTT	-	-	
			P, ATGGCCATCGGATCCTCAAYTCACTCT	CY5	BHQ2	
3	Influenza C	Matrix	F, GGCAAGCGACATGCTGAAYA	-	-	(2) Gunson et al, 2011
			R, TCCAGCTGCYTTCATTTGCTTT	-	-	
			P, CTCTTCCTTCTGATTTTTTCAAA	VIC	MGBNFQ	
4	HMPV A	Fusion	F, GCYGTYAGCTTCAGTCAATTCAA	-	-	(2) Gunson et al, 2011
			R, TCCAGCATTGTCTGAAAATTGC	-	-	
			P, CAACATTTAGAAACCTTCT	VIC	MGBNFQ	
5	HMPV B	Fusion	F, GCYGTYAGCTTCAGTCAATTCAA	-	-	(2) Gunson et al, 2011
			R, GTTATCCCTGCATTGTCTGAAAACT	-	-	
			P, CGCACAACATTTAGGAATCTTCT	VIC	MGBNFQ	
6	PIV-1	HN	F, GTGATTTAAACCCGGTAATTTCTCA	-	-	(2) Gunson et al, 2011
			R, CCTTGTTCCTGCAGCTATTACAGA	-	-	
			P, ACCTATGACATCAACGAC	FAM	MGBNFQ	
7	PIV-2	HN	F, ATGAAAACCATTTACCTAAGTGATGGA	-	-	(2) Gunson et al, 2011
			R, CCTCCYGGTATRGCAGTGACTGAAC	-	-	
			P, TCAATCGCAAAAGC	VIC	MGBNFQ	
8	PIV-3	HN	F, CCAGGGATATAYTAYAAAGGCAAAA	-	-	(2) Gunson et al, 2011
			R, CCGGGRCACCCAGTTGTG	-	-	
			P, TGGRTGTTCAAGACCTCCATAYCCGAGAAA	FAM	BHQ1	
9	PIV-4	Fusion	F, CAGAYAACATCAATCGCCTTACAAA	-	-	(2) Gunson et al, 2011
			R, TGTACCTATGACTGCCCCAAARA	-	-	
			P, CCMATCACAAGCTCAGAAATYCAAAGTCGT	CY5	BHQ2	
10	HCoV 229E	Nucleocapsid	F, CAGTCAAATGGGCTGATGCA	-	-	(2) Gunson et al, 2011
			R, AAAGGGCTATAAAGAGAATAAGGTATTCT	-	-	
			P, CCCTGACGACCACGTTGTGGTTCA	FAM	BHQ1	
11	HCoV OC43	Nucleocapsid	F, CGATGAGGCTATTCCGACTAGGT	-	-	(2) Gunson et al, 2011
			R, CCTTCCTGAGCCTTCAATATAGTAACC	-	-	
			P, TCCGCCTGGCACGGTACTCCCT	CY5	BHQ3	
12	HCoV NL63	1a gene	F, ACGTACTTCTATTATGAAGCATGATATTAA	-	-	(2) Gunson et al, 2011
			R, AGCAGATCTAATGTTATACTTAAAACTACG	-	-	
			P, ATTGCCAAGGCTCCTAAACGTACAGGTGTT	VIC	TAMRA	
13	RSV A	NP	F, AGATCAACTTCTGTCATCCAGCAA	-	-	(2) Gunson et al, 2011
			R, TTCTGCACATCATAATTAGGAG	-	-	
			P, CACCATCCAACGGAGCACAGGAGAT	FAM	BHQ1	
14	RSV B	NP	F, GCATCATTGCTGTCATTAACTCAATT	-	-	(3) Kamau et al, 2017
			R, GGTGTACCTCTRTACTCTCCCATTATG	-	-	
			P, TCAAGTGTGGTCYTAGGYAATGCAGC	VIC	TAMRA	
15	HRV	5'UTR	F, TGGACAGGGTGTGAAGAGC	-	-	(2) Gunson et al, 2011
			R, CAAAGTAGTCGGTCCCATCC	-	-	
			P, TCCTCCGGCCCCTGAATG	VIC	TAMRA	
16	Adv	Matrix	F, GCCACGGTGGGGTTTCTAAACTT	-	-	(2) Gunson et al, 2011
			R, GCCCCAGTGGTCTTACATGCACATC	-	-	
			P, TGCACCAGACCCGGGCTCAGGTACTCCGA	CY5	BHQ2	
17	*M. pneumoniae*	Cytoadhesin P1 gene	F, AAGCAGGAGTGACGGAAACAC	-	-	(2) Gunson et al, 2011
			R, CACCACATCATTCCCCGTATT	-	-	
			P, CTCCACCAACAACCTCGCGCCTA	CY5	BHQ2	

Abbrevations

FAM=6-carboxyfluorescein;BHQ= black hole quencher; NS=nonstructural; HN=hemagglutinin; NP=nucleoprotein; UTR=untranslated region; PIV=parainfluenza virus; HCoV=human coronavirus; RSV=respiratory syncytial virus; Adv=Adenovirus; HMPV=human metapneumovirus;

References

(1) 1: Gunson R, Maclean A, Davies E, Bennett S, Miller R, Carman WF. Development of a multiplex real-time RT-PCR that allows universal detection of influenza A viruses and simultaneous typing of influenza A/H1N1/2009 virus. J Virol Methods. 2010 Feb;163(2):258-61. doi: 10.1016/j.jviromet.2009.10.006. Epub 2009 Oct 23. PubMed PMID: 19854220.

(2) Gunson RN, Carman WF. During the summer 2009 outbreak of "swine flu" in Scotland what respiratory pathogens were diagnosed as H1N1/2009? BMC Infect Dis. 2011 Jul 13;11:192. doi: 10.1186/1471-2334-11-192. PubMed PMID: 21752259; PubMed Central PMCID: PMC3146830.

(3) Kamau E, Agoti CN, Lewa CS, Oketch J, Owor BE, Otieno GP, Bett A, Cane PA, Nokes DJ. Recentsequence variation in probe binding site affected detection ofrespiratory syncytial virus group B by real-time RT-PCR. J Clin Virol. 2017Mar;88:21-25. doi: 10.1016/j.jcv.2016.12.011. Epub 2017 Jan 5. PubMed PMID: 28107671; PubMed Central PMCID: PMC5331890.

Supplementary Table 2. List of all viral sequences identified with corresponding GenBank accession number and crude read deapth coverage.

Sample ID	Virus	Virus Abbreviation	Genome crude coverage*	Genome length	GenBank accession number
20692_1	Rhinovirus_A	HRV-A	3.441	6,822nt	MK989733
20692_1	RSVB	RSVB	253.603	15,287nt	MK989761
20692_3	Parvovirus_B19	B19	10.102	5,593nt	MK989716
20692_6	Rhinovirus_C	HRV-C	240.805	7,113nt	MK989748
20692_7	RVi_KEN_2B	RVi	13.758	9,770nt	MK989764
20692_18	Rhinovirus_C	HRV-C	586.337	7,121nt	MK989749
20692_20	RSVB	RSVB	20.176	15,262nt	MK989762
20692_25	Enterovirus_D68	EV-D68	105.957	7,261nt	MK989723
20692_31	Rhinovirus_C	HRV-C	8.626	6,651nt	MK989750
20692_33	Rhinovirus_B	HRV-B	9.832	7,059nt	MK989741
20692_39	Rhinovirus_C	HRV-C	11.381	7,107nt	MK989751
20692_42	HMPV	HMPV	19.71	13,234nt	MK989730
20692_43	Enterovirus_D68	EV-D68	24.48	7,251nt	MK989724
20692_44	Rhinovirus_A	HRV-A	53.015	7,166nt	MK989734
20692_48	Rhinovirus_B	HRV-B	21.809	6,879nt	MK989742
20692_51	Rhinovirus_C	HRV-C	5.99	7,021nt	MK989760
20692_52	Rhinovirus_A	HRV-A	28.979	7,118nt	MK989735
20692_52	Enterovirus_D68	EV-D68	9.101	7,328nt	MK989725
20692_55	Poliovirus	Polio	20.16	7,397nt	MK989713
20692_57	Rhinovirus_A	HRV-A	147.094	7,051nt	MK989736
20692_62	RSVB	RSVB	29.037	15,367nt	MK989763
20692_64	Bocavirus	HBoV	100.277	5,533nt	MK989727
20692_64	Rhinovirus_B	HRV-B	4.871	7,204nt	MK989743
20692_81	Rhinovirus_C	HRV-C	9.21	6,919nt	MK989752
20692_83	Rhinovirus_C_contig1	HRV-C	314.609	7,103nt	MK989753
20692_83	Rhinovirus_C_contig2	HRV-C	33.611	4,195nt	MK989754
20692_88	Coxsackievirus_A16	CV-A16	214.343	7,411nt	MK989714
20692_88	Enterovirus_D68	EV-D68	35.684	7,288nt	MK989726
20692_90	Rhinovirus_C	HRV-C	79.665	7,086nt	MK989755
20692_92	RVi_KEN_2B	RVi	5.678	9,588nt	MK989765
20692_93	Rhinovirus_C	HRV-C	151.153	7,151nt	MK989756
20692_95	Rhinovirus_C	HRV-C	14.438	7,103nt	MK989757
20693_1	Rhinovirus_A	HRV-A	5.934	7,087nt	MK989737
20693_9	Coxsackievirus_A24	CV-A24	6.018	7,326nt	MK989717
20693_11	Bocavirus	HBoV	11.358	5,497nt	MK989728
20693_14	HMPV	HMPV	7.99	13,303nt	MK989731
20693_16	Rhinovirus_A	HRV-A	42.407	6,866nt	MK989738
20693_21	Rhinovirus_B	HRV-B	3.24	6927nt	MK989744
20693_45	Rhinovirus_A	HRV-A	10.051	7,059nt	MK989739
20693_49	Coxsackievirus_A24	CV-A24	36.418	7,389nt	MK989718
20693_57	Rhinovirus_B	HRV-B	4.526	7,068nt	MK989745
20693_58	Rhinovirus_A	HRV-A	164.936	7,043nt	MK989740
20693_59	Rhinovirus_B	HRV-B	216.215	7,249nt	MK989746
20693_61	HMPV	HMPV	13.172	13,329nt	MK989732
20693_66	Coxsackievirus_A24	CV-A24	12.071	7,422nt	MK989719
20693_67	Coxsackievirus_A24	CV-A24	14.581	7,396nt	MK989720
20693_68	Human_parainfluenza_1	HPIV-1	232.83	15,560nt	MK989715
20693_74	Bocavirus	HBoV	9.595	5,512nt	MK989729
20693_82	Rhinovirus_B	HRV-B	13.571	7,186nt	MK989747
20693_83	Rhinovirus_C	HRV-C	143.663	7,135nt	MK989758
20693_84	Coxsackievirus_A24	CV-A24	32.712	7,477nt	MK989721
20693_86	Rhinovirus C	HRV-C	4.371	6965nt	MK989759
20693_94	Coxsackievirus_A24	CV-A24	31.566	7,307nt	MK989722

*Depth of sequence coverage was calculated as number of reads mapping with Bowtie2 [28] with " --very-sensitive-local" mapping to detect possible cross-talk sequences.

28. Langmead,B.&Salzberg,S.L. Fast gapped-read alignment with Bowtie2. Nat.Methods 9,357–359(2012).
